# Patient Gowns and Dehumanization During Hospital Admission: A Randomized Clinical Trial

**DOI:** 10.1001/jamanetworkopen.2024.49936

**Published:** 2024-12-10

**Authors:** Gehani Chamilka Punchihewa, Elizabeth Broadbent

**Affiliations:** 1 University of Auckland, Grafton, Auckland, New Zealand

## Abstract

This parallel-design double-blind randomized clinical trial examines the effects of patient gowns on feelings of dehumanization among individuals undergoing simulated hospital admission interviews in New Zealand.

## Introduction

Many hospitals require patients to change into hospital gowns during admission to the emergency department. Gowns can support infection control, examination, and treatment, but may not always be necessary. Observational studies suggest that gowns contribute to feelings of vulnerability, distress, disempowerment, and dehumanization, yet experimental research is lacking.^1,2^ This research aimed to experimentally investigate the effects of patient gowns on dehumanization.

## Methods

This study was approved by the Auckland Health Research Ethics Committee. The trial was registered at Australian New Zealand Clinical Trials Registry ACTRN12622000932763. All participants provided written informed consent (including audio recording). This report follows the CONSORT reporting guideline.

This parallel randomized clinical trial had an allocation ratio of 1:1 to wear a gown or own clothes. Inclusion criteria were age 18 years or older and speaking English. To allow for missing data, 74 participants were recruited between July 6, 2022, and August 24, 2022. Participants reported their ethnicity using options from the New Zealand census (classified by E.B.) to inform generalizability. The randomization sequence (stratified by gender) was generated by E.B. using an online research randomizer and hidden in sequentially numbered envelopes until the appointment. G.C.P. enrolled participants and assigned interventions, allowing participants to remain in their own clothes or asking them to change into a gown (eFigure in Supplement 1), then guided participants to another room for the interview with an intern. Participants underwent a standard hospital admission interview at a medical school clinic (medical history and vital signs check) matched by gender to either a male or female medical intern who wore usual work clothes. Common conditions included hypertension, hyperlipidaemia, type 2 diabetes, obesity, and asthma. Participants and interns were blind to the aim of the research and did not know that half were randomized to wear a gown. Interns were told that participants had the option to wear a gown. Participants’ speech during the consultation was recorded and analyzed for total words, emotion words, and singular first-person pronouns (indicating social status) using Linguistic Inquiry and Word Count software text analysis.^3,4^ After the consultation, participants completed the patient dehumanization questionnaire designed by the authors (primary outcome, eTable in Supplement 1) and provided written feedback on the consultation. G*Power software version 3.1.9.6 for Mac OS X 10.7 determined a sample size of 68 using power of 0.80, α of 0.05, and *d* of 0.70. Independent *t* tests were used in SPSS version 28.0.1.0 (IBM) to analyze questionnaires, speech, and blood pressure (indication of arousal). Two-tailed tests and a *P* value of .05 were maintained.

## Results

Among 74 participants, 37 (50%) were female and 37 (50%) were male; 40 (54%) were Asian, 3 (4%) were Māori or Pacific Islander, 27 (36%) were New Zealand European or European, and 4 (5%) were other race or ethnicity (African or Latin American); 61 (82%) were tertiary educated; and 42 (55%) had prior hospital admission. The Figure shows the CONSORT flow diagram. Participants wearing the gown reported feeling significantly more dehumanization than those wearing their own clothes (mean patient dehumanization questionnaire score for gown group: 23.47 [95% CI, 20.58-26.37] vs own clothes group: 18.03 [95% CI, 16.36-19.69]: *P* = .001), but there were no statistically significant differences in number of words spoken by participants or blood pressure (Table). Nine people wearing a gown mentioned the gown in their feedback, all in a negative way. For example, “Wearing a gown…made me feel uncomfortable and vulnerable…made me less likely to feel confident [to] speak up in an assertive manner” (patient 26, male). “Wearing the hospital outfit made me feel much more like a patient…” (patient 13, male).

**Figure.**
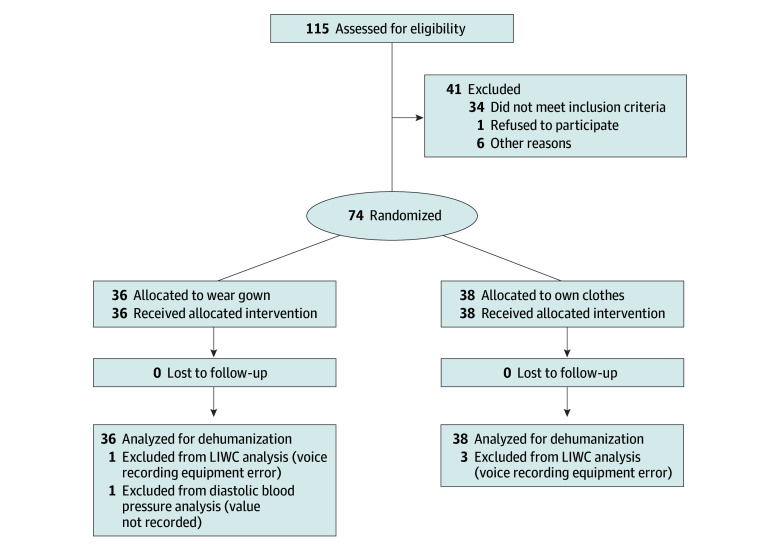
Flow of Participants Through Each Stage of the Randomized Clinical Trial LIWC indicates Linguistic Inquiry and Word Count software.

**Table.** Differences in Patient Dehumanization, Words Spoken, and Blood Pressure Between Participants Wearing Gowns vs Own Clothes

Outcome	Gown		Own clothes		Cohen *d*	*P* value
	No.	Mean (95% CI) [range]	No.	Mean (95% CI) [range]		
Patient dehumanization questionnaire	36	23.47 (20.58-26.37) [11-43]	38	18.03 (16.36-19.69) [11-30]	0.78	.001
Total word count	35	252.03 (200.72-303.34) [76-691]	35	330.17 (256.50-403.85) [78-864]	0.42	.08
First person personal pronoun use, %	35	9.29 (8.44-10.13) [4.03-13.11]	35	8.45 (7.59-9.31) [1.98-13.62]	0.34	.16
Negative emotion words, %	35	1.39 (1.04-1.75) [0-4.30]	35	1.45 (1.06-1.64) [0-3.85]	0.05	.84
Positive emotion words, %	35	3.79 (2.92- 4.67) [0.91-12.12]	35	3.94 (3.13- 4.75) [0-12.87]	0.06	.80
Systolic blood pressure, mmHg	36	119.61 (114.11-125.10) [97-153]	38	123.28 (116.95-129.62) [91-179]	0.2	.38
Diastolic blood pressure, mmHg	35	75.37 (72.13-78.61) [63-93]	38	79.55 (75.91-83.20) [59-120]	0.4	.09

## Discussion

This randomized clinical trial supports observational findings that patient gowns increase feelings of vulnerability and disempowerment.^1^ The lack of significant differences in speech could be due to the small sample size, and participants were positive about the interns’ professionalism. Blood pressure was influenced by medical conditions. Limitations include the clinical research setting with volunteers and the study did not aim to establish effects of gender or ethnicity. This research supports recommendations that patients be encouraged to wear personal clothing when gowns are not required.^5,6^

## Notes

Supplement 1. **eFigure.** The Hospital Gown Used in the Study **eTable.** Patient Dehumanisation Experiences Questionnaire Constructed by the Authors for the Study

Supplement 2.
Trial Protocol

Supplement 3.
Data Sharing Statement
