# Supplementary material for: Patient Gowns and Dehumanization During Hospital Admission: A Randomized Clinical Trial
Source: JAMA Netw Open. 2024 Dec 10;7(12):e2449936. doi: 10.1001/jamanetworkopen.2024.49936 (PMC11632538; doi:10.1001/jamanetworkopen.2024.49936)
Supplement: Supplement 1. — eFigure. The Hospital Gown Used in the Study eTable. Patient Dehumanisation Experiences Questionnaire Constructed by the Authors for the Study [file jamanetwopen-e2449936-s001.pdf]

Supplemental Online Content

Punchihewa GC, Broadbent E. Patient gowns and dehumanization during hospital admission: a randomized clinical trial. *JAMA Netw Open.* 2024;7(12):e2449936. doi:10.1001/jamanetworkopen.2024.49936

eFigure. The Hospital Gown Used in the Study

eTable. Patient Dehumanisation Experiences Questionnaire Constructed by the Authors for the Study

This supplemental material has been provided by the authors to give readers additional information about their work.

© 2024 Punchihewa GC et al. *JAMA Network Open.*

eFigure 1. The hospital gown used in the study.

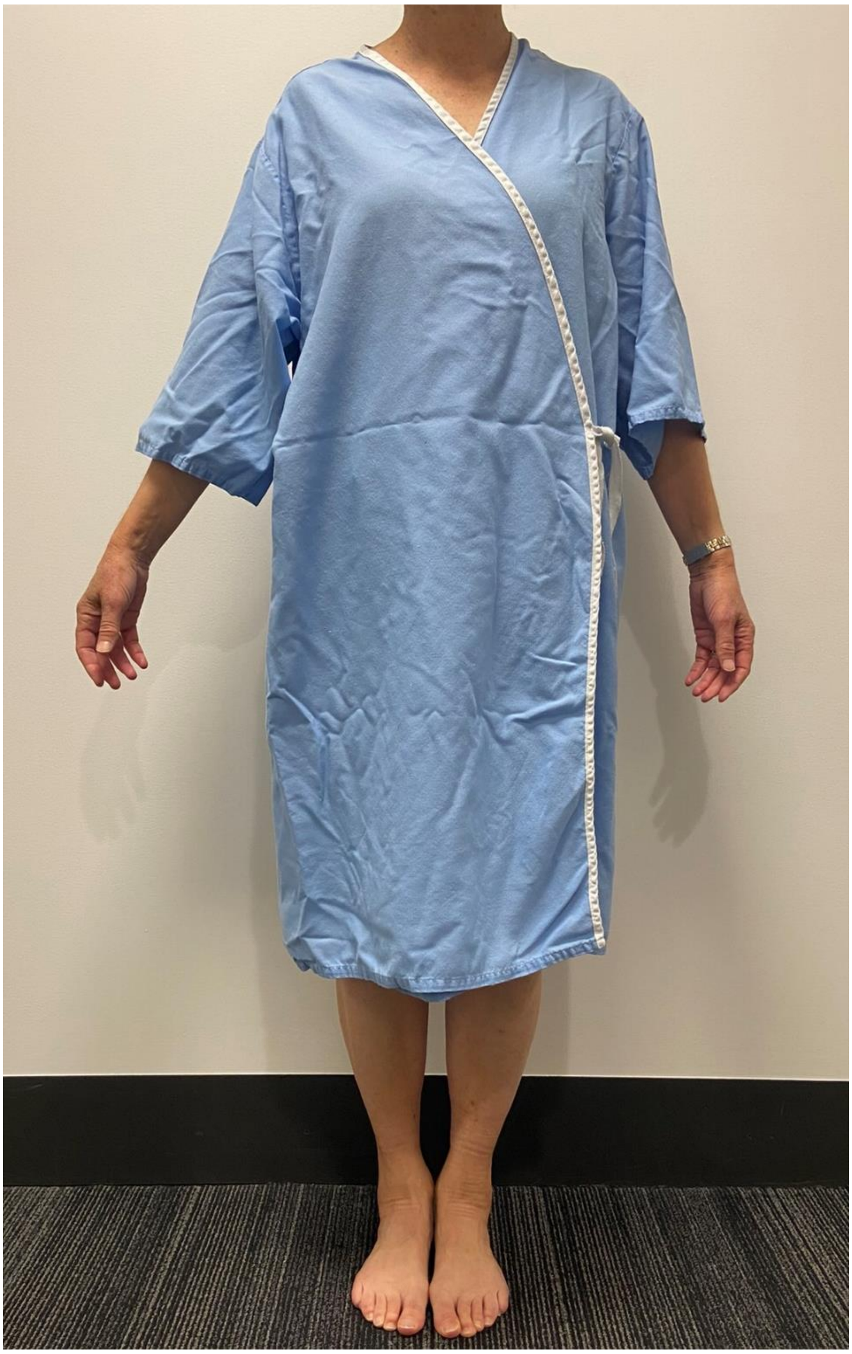

Note: Three sizes (large, medium, and small) of gowns were available. The gowns were designed as a wrap-around robe with external and internal ties. Participants were allowed to leave their underwear on underneath the gown.

© 2024 Punchihewa GC et al. *JAMA Network Open.*

eTable 1. Patient Dehumanisation Experiences Questionnaire constructed by the authors for the study

Please circle the appropriate number in the space next to each word to indicate the extent to which you felt this way **during the interview**.

**I felt . . .**	**Strongly Disagree**	**Disagree**	**Neutral**	**Agree**	**Strongly Agree**
Vulnerable	1	2	3	4	5
Confident*	1	2	3	4	5
Embarrassed	1	2	3	4	5
Anxious	1	2	3	4	5
Uncomfortable	1	2	3	4	5
Distressed	1	2	3	4	5
Low in self-esteem	1	2	3	4	5
In control*	1	2	3	4	5
Optimistic*	1	2	3	4	5
Restricted	1	2	3	4	5
Well*	1	2	3	4	5

*Item was reverse scored before summing. All items were summed for a total score. Scale Cronbach's alpha 0.91. Minimum total score 11, maximum total score 55. Higher scores represent greater feelings of dehumanisation.

© 2024 Punchihewa GC et al. *JAMA Network Open.*
