# Supplementary material for: Patient Gowns and Dehumanization During Hospital Admission: A Randomized Clinical Trial
Source: JAMA Netw Open. 2024 Dec 10;7(12):e2449936. doi: 10.1001/jamanetworkopen.2024.49936 (PMC11632538; doi:10.1001/jamanetworkopen.2024.49936)
Supplement: Supplement 2. — Trial Protocol [file jamanetwopen-e2449936-s002.pdf]

Patient Perceptions of Hospital Admission Interviews

Chamilka Punchihewa

Department of Psychological Medicine, The University of Auckland

Professor Elizabeth Broadbent

Patient Perceptions of Hospital Admission Interviews

Introduction

The recent emphasis on the provision of person-centred healthcare calls upon the health system and its service providers to recognise patients as human beings with complex needs, both psychologically and physically. Existing systems and processes within the health system often result in systematic dehumanisation of patients (Karan, 2019). By depersonalising the individual human experience via labelling the person as a patient with condition x, clothing them in revealing and anonymising patient hospital gowns, placing tags on their wrists and placing them in non-stimulating patient wards, people are psychologically prepped to take on the patient role, be vulnerable and to hand power over their care to their physicians (Haslam & Loughnan, 2014; Morton et al., 2020). This is especially true in the context of secondary or specialist care in New Zealand where patient hospital gown wearing, wrist tagging and provision of a bed, is standard practice upon admission. If New Zealand, like many other nations around the world, would like to improve the quality and experience of care provided in order to improve health outcomes, then experiences of dehumanisation in healthcare must be considered.

Dehumanisation has many operationalisations across various areas of research. In the context of healthcare and medicine, dehumanisation becomes important to consider as it highlights a situation in which patient agency and experience is impacted. Dehumanisation in healthcare takes place because systems and service providers fail to consider the mental states of its patients. Hence, dehumanisation in this context refers to mechanistic forms of dehumanisation rather than the malicious and animalistic treatment of patients by healthcare providers and systems (Haque & Waytz, 2012). Mechanistic dehumanisation according to Haslam (2006)'s Dual Model of Dehumanisation occurs more within interpersonal interactions and within organisational settings and are often a result of unconscious and

automatic processes. Those experiencing this form of dehumanisation will often be denied qualities of warmth, emotion, and individuality (Christoff, 2014; Haslam & Loughnan, 2014).

In treating people more as objects and less as humans, it is argued that people enter into “cognitive deconstructive” states, which are often embodied by emotional numbness, cognitive inflexibility, and thoughts with reduced clarity and meaning (Christoff, 2014). These cognitive states could lead to low mood and feelings of sadness. Further, people may accept a lower status and experience emotions of shame and guilt. In this way, experiencing mechanistic dehumanisation could have a significant negative impact on a person’s psychological health. Self-determination theory proposes that basic psychological needs such as competence, autonomy and relatedness must be met to be psychologically healthy (Ryan & Deci, 2000). When such needs are unmet, people are more likely to experience mental illnesses like anxiety, depression, and stress-related conditions (Christoff, 2014).

Research also highlights that dehumanisation of patients is more likely to result in provision of non-empathetic and non-compassionate care and can negatively impact patient outcomes (Halpern & Weinstein, 2004; Haque & Waytz, 2012; Haslam & Loughnan, 2014). Given scarce healthcare resources, system managers and providers are forced to do more with less and are constantly pushed to sacrifice humanising communication and behaviours such as taking time to learn about the patient’s family and what is important to them, in order to carefully manage scarce resources and reach funding targets. In such situations, the quality of the healthcare service is severely impacted by the systemic pressures placed on service providers. In addition to these systemic factors, routine procedures such as patient gown wearing has also been increasingly explored to understand its impact on experiences of dehumanisation for patients. This research protocol looks to further this area of research via the proposed study.

Patient gowns, especially in the hospital setting, were first introduced to better support infection control, for easy mobility, and to increase easy access to the patient’s body for the purposes of physical examination and undertaking medical and surgical procedures. Today, most hospital stays, as well as certain medical services such as imaging, will require patients to strip-off and wear a pre-worn backless gown. These gowns often do not accommodate different body shapes and sizes and are not designed to reflect the varying cultural beliefs and values which exist across diverse patient populations.

Research and theory highlights that what people wear is an expression of themselves, a self-representation, and an indicator to those around them about who they are and what matters to them (Tombs, 2011). Clothing helps people get into the role of who they want to be and how they want to be perceived. In this way, clothing is often argued to be a social tool supporting the person to feel confident and competent (Piacentini & Mailer, 2004). Adam and Galinsky (2012) propose that when considering the impact of clothing on psychological processes, both the clothing’s symbolic meaning and the physical experience of wearing it must be evaluated. Boddington et al. (2021)’s research across the United Kingdom supports this theory. In this study the authors looked at the experience of people with dementia wearing hospital attire. The results showed that people experienced distress after wearing hospital clothing, especially when considering how others perceived them. The study argues that clothing worn in the hospital has a significant impact on maintaining dignity, autonomy, and humanness.

Given the importance of clothing in maintaining one’s identity, self-confidence, and self-esteem, it is very important to consider the psychological impact of gown wearing for patients in healthcare settings. This is especially true if patient hospital gown wearing creates an experience of dehumanisation impacting health outcomes.

Research investigating the impact of patient hospital gowns on the dehumanising experiences in healthcare settings is scant. Most studies in this area have utilised qualitative (mostly interviews) or mixed methods (interviews and surveys) designs to understand both patient and service provider perspectives. For patients, their privacy and dignity are impacted as the hospital gowns often require assistance to wear properly. Even after assistance to dress, the gown’s backless design means they sometimes bare all as they walk the corridors of the hospital. The gown is often seen as a metaphor for illness, and once worn, invites the wearer to accept their lowered status as a patient and hand power over their mind and body to their carers as they take on the role of the sick and vulnerable (Jankovska & Park, 2019; Topo & Iltanen-Tähkävuori, 2010; Wellbery & Chan, 2014).

A study by Lucas and Dellasega (2020) showed that patients found gown wearing to be a disempowering process driven by provider needs. Providers in this study expressed guilt for putting accessibility concerns over patient comfortability and dignity. Further, providers shared that seeing patients in gowns provoked biases in the way they thought about the patients and their situations. This finding mirrors findings from other studies on perceptions patients have of healthcare providers and their attire, especially doctors wearing white coats. Patients perceive doctors wearing white coats to be of higher status and more powerful (Jennings et al., 2016). This research supports the theory that clothing has a direct impact on psychological processes for people in the context of healthcare.

Findings from previous research indicates that wearing a patient gown can indeed be a depersonalising and arguably a dehumanising experience. Whilst the gown serves important functions in the healthcare setting, the psychological impact of gown wearing must be considered if the health system is to provide more person-centred healthcare services to promote positive health outcomes.

The negative psychological effects of wearing a patient gown may impact physical health outcomes through behavioural and neuro-immune pathways. Research has shown direct links between stress, immune function, behaviour, physical health, and mental health outcomes (Tausk et al., 2008; Zachariae, 2009). Hence, if patients are feeling vulnerable and lack self-confidence, this could impact their overall health and wellbeing. For instance, a person admitted following a fall may feel more fragile and vulnerable in the hospital gown. This could impact how well they do on assessments for discharge and how strong and safe they feel to go back home.

The patient gown can impact the doctor-patient relationship as highlighted previously. Patients take on a lower status and accept carers as more powerful once gowned, and physicians are unable to gather any personal information from patient attire about who the person in front of them is. Hence, both patient and provider behaviour may be impacted and reflected in overall patient outcomes (Morton et al., 2020). It is also interesting to note that some research has also identified that gown wearing is not always required for certain healthcare contexts, yet patients are still requested to change into gowns. This highlights the need to re-evaluate routine practices in some contexts (ref).

Given the very limited research exploring the impact of hospital gowns on dehumanising experiences in healthcare, this study hopes to extend what is known via conducting experimental research in this area that could allow conclusions around causation to be made more confidently. Using an experimental study design, this research will investigate the extent to which patient attire, specifically the hospital patient gown, contributes to the dehumanisation experience of patients. It is hoped that this study will help raise awareness of the effects gown wearing has on patient psychology.

As health systems around the world move towards a more person-centred model of healthcare, it is important to consider psychological enablers and barriers to health. Exploring the impact of patient attire has largely been ignored and thus deserves more attention. Though it may seem basic, as highlighted previously, what one wears can impact one's identity, mental health and ultimately health and wellbeing outcomes. As well as raising awareness about the impact patient attire has on patient healthcare experiences, this research hopes the findings will support providers to reflect on wider everyday practices within the services they provide and ask questions about whether they are facilitating a health promoting environment where patients' needs guide their decisions and actions.

Aims and Hypotheses

This study aims to investigate the impact of wearing a patient hospital gown on the patient experience, specifically focusing on dehumanisation. The study will also look at the impact of wearing a patient gown on the patient-doctor relationship, and patient behaviour.

Given previous research findings, it can is hypothesised that the participants wearing patient hospital gowns will report more thoughts and emotions around the loss of identity, power, and dignity as well as greater identification with the sick role, compared to those participants wearing their usual clothes.

It is also hypothesised that participants wearing gowns will behave in similar ways to a subordinate, relinquishing more power to the physician, than participants wearing their usual clothes, as measured by the language used by the participants. Physiological levels of arousal in participants wearing a patient gown may be higher than those of participants in their usual clothes, as measured through blood pressure readings.

Method

Study Design

The study will be a randomised controlled trial in which participants will be allocated to either wear a patient gown or their usual clothes before participating in an hospital admission interview with a doctor. The study will be pre-registered with the Australian New Zealand Clinical Trial Registry.

Eligibility Criteria

To be included in the study participants will have to be at least 18 years of age or older, and be able to speak, read and write in English. Participants will be required to attend the hospital admission interview in-person at The Auckland University Grafton Campus. Individuals requiring a support person to attend the admission interview with them will be excluded from the main analysis, since the presence of a support person may impact outcomes.

Study Outcomes

The primary outcome of this study will be the level of dehumanisation experienced by the participant following the admission interview. This will be measured via administration of a questionnaire. The questionnaire has been developed by the investigators of this proposed study, named the "The Patient Experience and Feedback Questionnaire," to measure the presence of factors proposed to contribute to the experience of dehumanisation for patients in the healthcare setting.

The secondary outcomes of this study will be the impact on patient behaviour. Specifically, the language participants use during the interview to communicate with their doctor will be analysed. To do this, the interview audio will be recorded, transcribed, and used with the Linguistic Inquiry and Word Count (LIWC) software. The doctor will measure patient vital

signs as part of the standard admission history taking and examination process. The blood pressures of participants taken during the interview will be looked at as a secondary outcome.

Sample Size

A power analysis was carried out using G*Power software with a power level of .80 and a two-tailed significance level of α =.05, with an effect size of d = 0.70. Using these parameters gives a required total sample size of 68. The lack of previous experimental data on gown wearing made an estimation of expected effect size more challenging. Research on the effects of submissive posture on self-esteem and first-person pronouns has demonstrated effect sizes of d = 0.79, and d = 1.16 (Nair et al., 2015). Given the study is cross-sectional, attrition does not need to be accommodated, but we will recruit 70 participants in case of missing data.

Recruitment

70 participants will be recruited from within The University of Auckland via flyers, student email lists, online advertising, and in-lecture announcements. To ensure fair representation of all ethnic groups in the study, coordinators of the Māori and Pacific Admission Scheme (MAPAS) and Tuākana programs will be contacted to prioritise the inclusion of Māori and Pacific participants proportional to the NZ population. Those who respond to the advertisement will be sent a participant information sheet and checked to see if they meet the eligibility criteria before enrolling in the study. Once enrolled in the study, participants will be contacted via email with details about the experiment dates, times, and locations. Written consent of participants to partake in the study will be gained upon arrival at the study venue.

Study Procedure

The randomised control trial will be a single-centre study conducted at The University of Auckland Grafton Campus. The 70 participants for the trial will be recruited at The

University of Auckland via online, email, and poster advertisements. The participants will be told that the study is looking to evaluate two different types of medical interviews and that they will be supporting the researchers in this evaluation via the provision of feedback.

Once the potential participants respond to the advertisements for the study via email, a participant information sheet with study details will be sent to them. Upon meeting eligibility criteria participants will be asked to register via an online booking system to attend a medical interview at a date and time convenient to them. Participants will have a selection of dates and times available to choose from.

Once participants arrive at the research venue, they will be asked to read and sign a written consent form and be given the opportunity to ask any questions they may have about the study. Participants will also complete a baseline questionnaire collecting demographic details. Once paperwork is complete, participants will be randomised to either wear a patient hospital gown or remain in their own clothes. The researcher completing the pre-interview process with participants will be blinded to the randomisation process and will only know which group the participant will be in after opening a sealed envelope with the information post pre-interview procedures. The randomisation will be completed using randomiser.org by the second researcher who will not be present at the research venue during the interviews.

After randomisation of the participants to either the gown wearing or wearing own clothes group, participants will enter the medical interview room to meet the doctor and begin the interview. The medical interviews will be carried out by either a female or male 5th or 6th year medical student from The University of Auckland. The medical students will be employed by the research team to conduct the interviews. The “doctors” too will be told that they are supporting a study looking to evaluate two different types of medical interviews. Their role will be to conduct the interview following a script provided by the researchers. The medical

students will be asked to wear what they would normally wear for hospital ward rounds. They will also be asked to sign a confidentiality statement requiring them not to discuss the study, the participants or share their thoughts about the interview styles with others and each other. The medical interview will follow a standard interview structure and finish by taking the vital signs (Lichstein, 1990). Participants will receive either the female or male doctor depending on which date and time they book their interview for via the online booking system. The interview times available on the online booking system will reflect availability of the employed medical students and the researcher.

During the medical interview, participants will be asked to answer questions truthfully. Participants will not have a specific presenting complaint to share with the doctor. If the "doctors" have any concerns about the health and wellbeing of the participants following the interview, they will be asked to share these with the researcher who will be able to take the necessary steps to ensure the participant is followed-up, informed and given an opportunity to seek professional support.

During the interview, the conversation between the participant and doctor will be recorded using a voice recorder. This data will later be transcribed and analysed using LIWC post-interview. The blood pressure of participants will also be measured as part of taking the vital signs. After completing the interview with the doctor, participants will be asked to complete The Patient Experience and Feedback Questionnaire.

It is expected that the participants will spend a total of 40 minutes at the clinical research centre. This will include 10 minutes upon arrival spent gowning up if required and completing the baseline questionnaire. The patient-physician consultation is expected to take no more than 20 minutes followed by 10 minutes to complete the post-interview

questionnaires. Participants will receive a $20 Countdown voucher as a gesture of gratitude for volunteering their time for the study as they leave the venue.

Measures

Baseline Questionnaire

Participants will be given a demographics questionnaire which includes questions regarding age, ethnicity, gender, previous experiences with hospital admissions and the degree if completing a university study. Collecting data on these variables will allow for subgroup analysis and comparisons of the groups at baseline.

Primary Outcome

Patient Experience and Feedback Questionnaire

This questionnaire looks to identify how the participants felt, their thoughts and emotions during the medical interview. The questionnaire was developed by the researchers based on proposed factors contributing to dehumanising experiences in healthcare settings. There are currently no other questionnaires available that measure experiences of dehumanisation in the context of healthcare.

Secondary Outcomes

Language Analysis

The language used by participants during the interviews will be analysed using LIWC's student -academic version. Audio recorded language will be transcribed and entered to LIWC. LIWC will read and compare each word in the text to the list of dictionary words and

calculate the percentage of total words int eh text that match each of the dictionary categories. The study will look at the use of pronouns and positive/negative language.

Blood Pressure

Blood pressure will be measured as an indicator of physiological arousal. The measure will be taken as part of the vital signs during the interview by the doctor.

Statistical Analysis

IBM SPSS software will be used to analyse quantitative data collected from the trial. Independent t tests will be used to identify differences between the two groups for self-report measures and blood pressure. ANCOVA analysis will be conducted to control for covariates known to affect outcomes.

All results obtained will be considered statistically significant if p-values < .05. The results will be analysed and reported as described in the consolidated standards of reporting trial (CONSORT) guidelines to maintain transparency.

Ethics

The study will only commence after receiving ethics approval from The Auckland Health Research and Ethics Committee. The study participants will be provided with a detailed participant information sheet and an opportunity to ask any questions and clarify any points prior to enrolling in the study. They will also be encouraged to seek external support if required when making the decision to partake in the research. Informed consent will be obtained prior to study enrolment and participants will sign a consent form agreeing to volunteer for the study upon arrival at the research venue for their interview. To ensure

anonymity and confidentiality of participant data, each participant will be given a unique participant identifier number. All data collected about each participant will be stored in secure password-protected files in encrypted servers. In case of any unexpected findings (e.g., hypertension), the participant will be informed and advised to seek advice from their general practitioner or student health services.

References

Adam, H., & Galinsky, A. D. (2012). Enclothed cognition. *Journal of Experimental Social Psychology*, *48*(4), 918-925. https://doi.org/https://doi.org/10.1016/j.jesp.2012.02.008

Boddington, P., Featherstone, K., & Northcott, A. (2021). Presentation of the clothed self on the hospital ward: an ethnographic account of perceptual attention and implications for the personhood of people living with dementia. *Medical Humanities, 47*(2), e3. https://doi.org/10.1136/medhum-2019-011757

Christoff, K. (2014). Dehumanization in organizational settings: some scientific and ethical considerations. *Frontiers in human neuroscience*, *8*, 748-748. https://doi.org/10.3389/fnhum.2014.00748

Halpern, J., & Weinstein, H. M. (2004). Rehumanizing the Other: Empathy and Reconciliation. *Human Rights Quarterly, 26*(3), 561-583. http://www.jstor.org/stable/20069745

Haque, O. S., & Waytz, A. (2012). Dehumanization in Medicine:Causes, Solutions, and Functions. *Perspectives on Psychological Science, 7*(2), 176-186. https://doi.org/10.1177/1745691611429706

Haslam, N. (2006). Dehumanization: An Integrative Review. *Personality and Social Psychology Review, 10*(3), 252-264. https://doi.org/10.1207/s15327957pspr1003_4

Haslam, N., & Loughnan, S. (2014). Dehumanization and Infrahumanization. *Annual Review of Psychology*, *65*(1), 399-423. https://doi.org/10.1146/annurev-psych-010213-115045

Jankovska, D., & Park, J. (2019, 2019/05/04). A mixed-methods approach to evaluate fit and comfort of the hospital patient gown. *International Journal of Fashion Design,*

Technology and Education, *12*(2), 189-198. https://doi.org/10.1080/17543266.2018.1551942

Jennings, J. D., Ciaravino, S. G., Ramsey, F. V., & Haydel, C. (2016). Physicians' Attire Influences Patients' Perceptions in the Urban Outpatient Orthopaedic Surgery Setting. *Clinical Orthopaedics and Related Research®*, *474*(9), 1908-1918. https://doi.org/10.1007/s11999-016-4855-7

Karan, A. (2019). The dehumanisation of the patient. *BMJ*, *367*, l6336. https://doi.org/10.1136/bmj.l6336

Lichstein, P. R. (1990). The Medical Interview. In H. K. Walker, W. D. Hall, & J. W. Hurst (Eds.), *Clinical Methods: The History, Physical, and Laboratory Examinations* (3 ed.). Butterworths

https://www.ncbi.nlm.nih.gov/books/NBK349/

Lucas, C. M., & Dellasega, C. (2020). Finding common threads: How patients, physicians and nurses perceive the patient gown. *Patient Experience Journal*, *7*(1), 51-64. https://doi.org/10.35680/2372-0247.1387

Morton, L., Cogan, N., Kornfält, S., Porter, Z., & Georgiadis, E. (2020). Baring all: The impact of the hospital gown on patient well-being. *British Journal of Health Psychology*, *25*(3), 452-473. https://doi.org/https://doi.org/10.1111/bjhp.12416

Nair, S., Sagar, M., Sollers, J., 3rd, Consedine, N., & Broadbent, E. (2015). Do slumped and upright postures affect stress responses? A randomized trial. *Health Psychol*, *34*(6), 632-641. https://doi.org/10.1037/hea0000146

Piacentini, M., & Mailer, G. (2004). Symbolic consumption in teenagers' clothing choices. *Journal of Consumer Behaviour, 3*(3), 251-262. https://doi.org/https://doi.org/10.1002/cb.138

Ryan, R. M., & Deci, E. L. (2000). Self-determination theory and the facilitation of intrinsic motivation, social development, and well-being. *American psychologist*, *55*(1), 68.

Tausk, F., Elenkov, I., & Moynihan, J. (2008). Psychoneuroimmunology. *Dermatol Ther*, *21*(1), 22-31. https://doi.org/10.1111/j.1529-8019.2008.00166.x

Tombs, A. (2011). Do our feelings leak through the clothes we wear? *Australian and New Zealand Marking Academy*.

Topo, P., & Iltanen-Tähkävuori, S. (2010, 2010/06/01/). Scripting patienthood with patient clothing. *Social Science & Medicine*, *70*(11), 1682-1689. https://doi.org/https://doi.org/10.1016/j.socscimed.2010.01.050

Wellbery, C., & Chan, M. (2014). White coat, patient gown. *Medical Humanities, 40*(2), 90-96. https://doi.org/10.1136/medhum-2013-010463

Zachariae, R. (2009). Psychoneuroimmunology: A bio-psycho-social approach to health and disease. *Scandinavian Journal of Psychology, 50*(6), 645-651. https://doi.org/https://doi.org/10.1111/j.1467-9450.2009.00779.x
