# Supplementary material for: Patient Gowns and Dehumanization During Hospital Admission: A Randomized Clinical Trial
Source: JAMA Netw Open. 2024 Dec 10;7(12):e2449936. doi: 10.1001/jamanetworkopen.2024.49936 (PMC11632538; doi:10.1001/jamanetworkopen.2024.49936)
Supplement: Supplement 3. — Data Sharing Statement [file jamanetwopen-e2449936-s003.pdf]

Data Sharing Statement

Punchihewa. Patient Gowns and Dehumanization During Hospital Admission. *JAMA Netw Open*. Published December 10, 2024. doi:10.1001/jamanetworkopen.2024.49936

Data
Additional Information: at Australian New Zealand Clinical Trials Registry ACTRN12622000932763 https://www.anzctr.org.au/
Data available: No

Additional Information
Explanation for why data not available: Participant data is confidential according to our ethics approval conditions
